# Dr. O. N. Allan

**Published:** 1875-07

**Authors:** 


### OBITUARY.

*Dr. O. N. Allan.*—A letter from Danville, Va., dated June 21st, 1875, says:—"This community was greatly shocked on Sunday night last upon their hearing of the sudden death of Dr. Allan, who expired immediately after tea. It appears that he took tea in his usual good health, and was conversant with his family and others up to that time, and immediately after leaving the dining room, went up stairs, leaving the rest of the family at the table, fell and almost immediately expired. Dr. A. had been upon the street in the forenoon, conversing as usual with his friends, and did not express or show any ailment whatever. It is supposed that he died with heart disease. "Truly in the midst of life we are in death."

"Dr. Allan has been a resident of this town for the last twenty-one years, and was a successful practioner in dentistry, was a useful and good citizen, and beloved by all who knew him, and his death is much regretted by the whole community."

Dr. Allan about 25 years ago removed to Danville, where he has lived ever since, with the exception of about two years he spent at Culpeper. All who knew him—and there are many—loved him as a neighbor and friend, and for the goodness of heart. He was aged sixty-five years.

